# The Effect of Reduced Water Availability in the Great Ruaha River on the Vulnerable Common Hippopotamus in the Ruaha National Park, Tanzania

**DOI:** 10.1371/journal.pone.0157145

**Published:** 2016-06-08

**Authors:** Claudia Stommel, Heribert Hofer, Marion L. East

**Affiliations:** Leibniz Institute for Zoo and Wildlife Research, Alfred-Kowalke-Strasse 17, 10315, Berlin, Germany; U.S. Geological Survey, UNITED STATES

## Abstract

In semi-arid environments, ‘permanent’ rivers are essential sources of surface water for wildlife during ‘dry’ seasons when rainfall is limited or absent, particularly for species whose resilience to water scarcity is low. The hippopotamus (*Hippopotamus amphibius*) requires submersion in water to aid thermoregulation and prevent skin damage by solar radiation; the largest threat to its viability are human alterations of aquatic habitats. In the Ruaha National Park (NP), Tanzania, the Great Ruaha River (GRR) is the main source of surface water for wildlife during the dry season. Recent, large-scale water extraction from the GRR by people upstream of Ruaha NP is thought to be responsible for a profound decrease in dry season water-flow and the absence of surface water along large sections of the river inside the NP. We investigated the impact of decreased water flow on daytime hippo distribution using regular censuses at monitoring locations, transects and camera trap records along a 104km section of the GRR within the Ruaha NP during two dry seasons. The minimum number of hippos per monitoring location increased with the expanse of surface water as the dry seasons progressed, and was not affected by water quality. Hippo distribution significantly changed throughout the dry season, leading to the accumulation of large numbers in very few locations. If surface water loss from the GRR continues to increase in future years, this will have serious implications for the hippo population and other water dependent species in Ruaha NP.

## Introduction

Human utilisation of water resources is increasing worldwide and is likely to accelerate given the projected growth in the human population [1], and the increasing volume of water required for domestic, agricultural and industrial purposes [2, 3]. This growing demand on water resources is likely to have potentially negative consequences for ecosystems and the wildlife they support.

In regions of the world that experience ‘dry seasons’ when little or no precipitation falls for several months, seasonal rivers and other sources of surface water typically dry up soon after the onset of the dry season, hence sources of surface water are scarce. ‘Permanent’ rivers that persist throughout the dry season provide a vital source of water to the areas through which they flow. Human extraction of water from permanent rivers can result in a decrease in dry season flow and the absence of surface water during dry seasons. This may have negative consequences for wildlife populations that depend on water from permanent rivers during dry seasons, yet current knowledge on the resilience of many species to anthropogenic restrictions of water resources is limited.

Historically the common hippopotamus (*Hippopotamus amphibius*), hereafter termed the hippo, was once widely distributed throughout sub-Saharan Africa. Its range has declined and become fragmented and its population size has contracted chiefly as a result of human activities [4]. Since 2006, the hippo has been listed by IUCN as a threatened species, vulnerable to extinction (www.iucn-redlist.org). One of the main threats to this species is habitat loss caused by human conversion of wetlands to agricultural land and the redirection of water from rivers and lakes to agricultural areas (e.g. [5–7]). Hippo populations may crash in drought years because of reduced conception/fertility and increased mortality associated with heat stress, poor nutrition and an increased vulnerability to diseases, as large numbers of animals congregate in declining water sources [6, 8]. Although some climate models predict a future increase in rainfall for East Africa, there has been a downward trend in rainfall in the region since the 1980s [9–11]. If this trend continues it is likely to negatively affect hippo populations.

Hippos are particularly vulnerable to changes in their aquatic environment because they require regular daytime submersion in water to prevent skin damage by the sun and to help body temperature regulation [4, 12]. Their skin is to some extent protected from sun damage by a viscous fluid produced by sub-dermal glands that turns red-brown after secretion and also has antimicrobial properties [13]. At night, hippos leave their daytime resting areas and may move several kilometres to their grazing areas [14]. A recent stable isotope study indicates that hippos consume browse [15] and hence may not be the strict grazers they were previously assumed to be [4]; they are also opportunistically carnivorous [16].

In areas containing permanent water, hippo bulls may hold a territory for many years and in some cases throughout adult life (~ 20 years or more). Territory ownership is advertised by ritualized defecation, urination and dominance behavioural displays. Contests between neighbouring bulls are normally ritualized but fights can result in serious wounds [17]. Mating occurs in water; territorial males are thought to monopolise mating access to receptive females in their territory [17]. Non-territorial males may join bachelor herds and are tolerated inside the territories of bulls provided they display submissive behaviour [4, 17]. Adult females normally establish a home range in their natal area that encompasses the territory of more than one bull. Females give birth in water to a single offspring that can suckle even under water [12].

When their day resting areas dry out, hippos may travel beyond their territory and normal range to locate alternative suitable daytime resting sites [18]. This disrupts established relationships among hippos in an area and increases the probability of interactions with less familiar hippos [19] and hence the chance of intra-specific aggression [19, 20, 21, 22]. Females may encounter sexual harassment [17] and calves may be killed by infanticidal bulls that aim to bring their mothers into oestrus [23]. Film footage of a subadult hippo committing infanticide also suggests that the disruption of normal relationships and physiological stress associated with high hippo densities in some day resting areas may also precipitate infanticide [17].

The Great Ruaha River (GRR) is an important permanent river in central Tanzania that flows through Ruaha National Park (NP) where it is the main source of surface water for wildlife, especially during the dry season [24, 25]. It is thought that during the dry season most hippos in the Ruaha ecosystem reside in Ruaha NP and probably most are confined to the GRR [25]. Large scale human utilization of water from the GRR upstream of Ruaha NP since 1993 is considered chiefly responsible for a significant reduction in the dry season flow and the loss of surface water from extensive stretches of the GRR within Ruaha NP [24]. The reduced dry season flow of water also results in a significant reduction in water quality, in terms of increased salinity, aerobic bacterial load and faecal contamination [25]. The impact of the reduced flow and quality of surface water, and the decline in the availability of surface water in the GRR during the dry season on the distribution of hippos has not been investigated. Knowledge on the current impact of reduced water flow on hippos is important and can serve as a benchmark for future studies.

Our study focused on daytime resting sites used by hippos along a substantial section of the GRR within the Ruaha NP. We expected changes in hippo distribution during the dry season to be driven by a decline in surface water and water quality, leading to a reduction in suitable daytime resting sights. If hippos have to abandon unsuitable daytime resting locations and relocate to another suitable location, this should result in the congregation of hippos at a few key locations as the dry season progresses. We aimed to identify whether the same key daytime resting areas are used across years and expected hippo mortality associated with poor nutrition, intra-specific aggression and disease to be lower in the early than the late dry season. We discuss the implication of our findings in relation to the long-term prospects for the GRR population of hippos within Ruaha NP.

## Methods

### Study area

**Fig 1:**
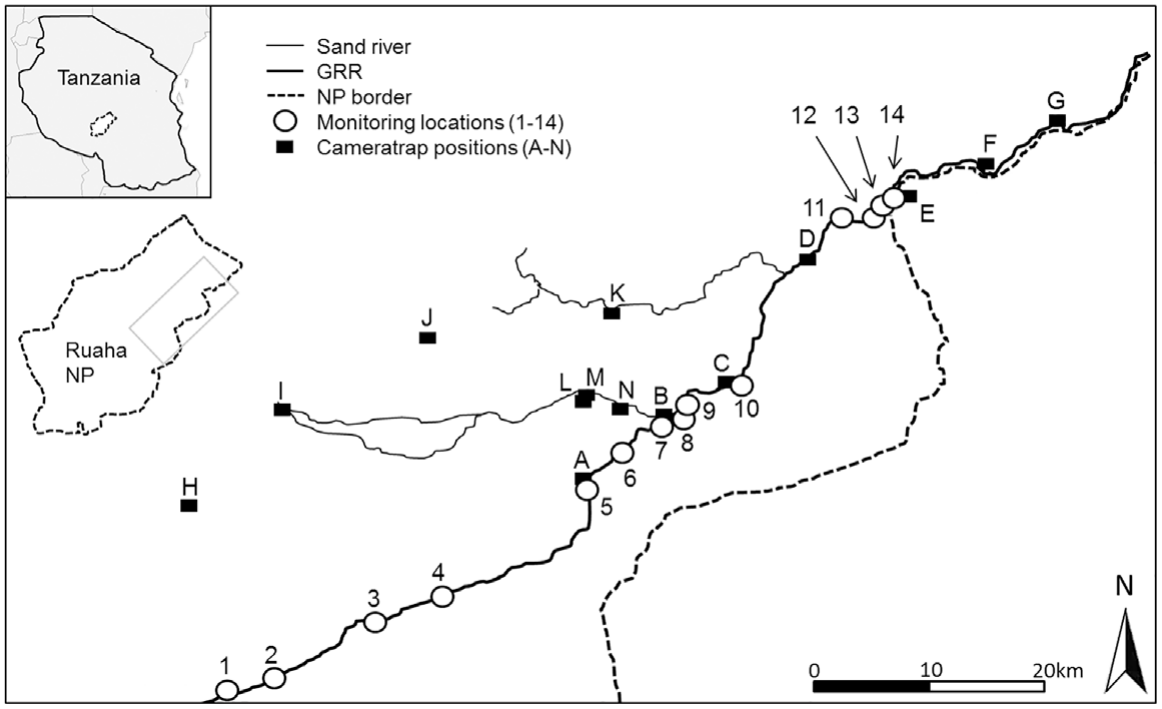
The Ruaha National Park in central Tanzania with the study area in the east (grey square frame). Monitoring locations (circles) were located along the Great Ruaha River (GRR, sites 1–14). The camera traps (black square boxes) were located along the GRR (A-G) and at non-GRR water sources, sites (H-N). The map was created using ArcGIS and includes a modified outline of rivers derived from “Diva-GIS” [42] and a modified outline of the border of the National Park derived from “The World Database on Protected Areas” [43].

Our study took place in the Ruaha NP in central Tanzania, the largest (20,226 km^2^) National Park in East Africa (Fig 1). Data were collected during the 2012 and 2013 dry seasons, both of which spanned a period of six months (June to November). The RNP receives a mean annual rainfall of approximately 580mm which almost exclusively falls during the wet season [26]. During the 2013 dry season negligible precipitation occurred in a few local showers (< 6mm) that evaporated within a few hours. In November 2012, one rainstorm delivered 52mm of rain which briefly increased surface water availability in the study area [25].

During the 2012 and 2013 dry season, large sections of the GRR dried out, leaving discrete water pools separated by stretches of dry river bed [25]. Tributaries of the GRR that flowed in the wet season became dry sand-rivers with occasional, localised pools of water during these dry seasons. Springs at some distance northwest of the GRR also contained relatively small amounts of surface water [25].

### Censusing the distribution and numbers of hippos

Previous censuses of the Greater Ruaha ecosystem, encompassing 43.601 km [27, 28] and including Ruaha NP, Rungwa, Kizigo and Muhesi Game Reserves demonstrated that the distribution of hippos is limited to the GRR and Mzombe River and that the majority occurred in the GRR inside Ruaha NP. We therefore censused the distribution of hippos inside Ruaha NP at 14 locations (from now on called monitoring locations) that were used by hippos for daytime resting (locations 1 to 14, Fig 1). The topographical gradient of the GRR changed across the study site. The elevation of the river declined by 156 m along the 53.5 km covered between monitoring location 1 to camera trap position B (Fig 1), whereas it changed only 43 m over a distance of 50.5 km between camera trap positions B and G (Fig 1). Hence, we categorised the topographic gradient between locations 1 and B as “upstream” and “steep” and the gradient between locations B and G as “downstream” and “flat”.

All monitoring locations could be viewed from the game viewing roads along the northern bank of the GRR. We aimed to count hippos at these 14 monitoring locations during censuses twice per month but, this was not always feasible. Twelve out of 14 monitoring locations were censused once in November, locations 5 in 2012 and 8 in 2013 were not monitored. As it was not possible to count hippos at all 14 locations along the GRR within one day, the river was divided into three censusing sections. Hippos were counted in two sections between monitoring locations 1 and camera trap position B (Fig 1) on two consecutive days; those in the section between camera trap position B and monitoring location 14 (Fig 1) were counted on one day. Counts of all 14 monitoring locations were completed within a mean of 4.41 ± 0.23 successive days (SEM).

Hippos were counted by scanning each monitoring location for at least seven minutes using binoculars. This minimum observation period was used because hippos can stay submerged for up to seven minutes [29]. Even so, some counts are likely to be minimum estimates of the number of hippos present, particularly in large congregations when juveniles may have been overlooked. When feasible, hippos were also sexed and allocated to three age categories: infants (estimated age < one year), juveniles (estimated age from > one year to not yet adult) and adults (fully grown animals). We defined counts from June to August as early dry season counts and those from September to November as late dry season counts. To illustrate changes in hippo distribution in greater detail we focused on two upstream monitoring locations (1 and 4, Fig 1) and two downstream monitoring locations (9 and 11, Fig 1) which we have censused most frequently throughout both dry seasons. The GPS coordinates for these four monitoring locations are: 1 = 674821.00 mE, 9125948.00 mS; 4 = 693486.00 mE, 9134339.00 mS; 9 = 714169.00 mE, 9150895.00 mS; 11 = 727870.00 mE, 9166962.00 mS (UTM zone 36 M).

### The state of water expanse at monitoring locations

The monitoring locations (except no. 12 in 2012 and 2013, and no. 8 in 2013) were categorised every two weeks in terms of the estimated depth and expanse of water they contained. The status categories were: (1) dry—no water present, (2) shallow—an amount of water to rest in throughout one day that is inadequate for an adult hippo (< approximately 10 cm deep), (3) moderate—an expanse of water (>0.5m deep) assessed to be sufficient to accommodate approximately 15 resting hippos, (4) large—an expanse of water (>0.5m deep) sufficient to accommodate more than 20 hippos.

### Water quality

Water quality was measured in terms of salinity and total aerobic bacterial load. Water salinity was measured for water samples collected every two weeks from June to November at ten sampling points along the GRR as previously described in detail [25]. Briefly, salinity was measured in micro Siemens per centimetre (μS/cm) using a Multi 340i Multimeter (Wissenschaftlich Technische Werkstätten GmBH, Weilheim, Germany). Total aerobic bacterial load was measured as the number of colony forming units per ml (cfu/ml) using the 3M Petrifilm Aerobic Count Plate including a tetrazolium indicator (*3M*^™^ *Petrifilm*^™^ *Aerobic Count Plates* (U.S. AOAC^®^)), following the manufacturer’s instructions—for details see [25].

Water flow was categorised as “dry” (no surface water), “stagnant” (non-flowing surface water) and “flowing” (moving surface water) twice every month at these ten water sampling points—for details see [25].

### Camera trapping

We used Reconyx PC800 camera traps (Reconyx Inc., Holmen, Wisconsin, USA) to record the presence of hippos at seven sites along the GRR (camera trap positions A-G, Fig 1), at five locations with surface water in dry sand rivers (camera trap positions I, K-N, Fig 1) and at two springs (camera trap positions H and J, Fig 1) from August to November in 2012 and from June to November in 2013. One camera monitored each location for a mean of 960.2 ± 36.3 h in 2012 and a mean of 1473.0 ± 86.3 h in 2013. Total monitoring time was 13,443.1 h in 2012 and 20,622.1 h in 2013. A total of 205,959 camera pictures were analysed. To define an independent “hippo camera trapping event” we set a minimum period of 15 minutes between the last picture of the previous hippo trapping event and the first picture of the next hippo trapping event.

### Transect observations

The distribution of larger mammals, including hippos, was recorded along ten ground transects, each 20km in length, from June to November in 2012 and 2013. Transects were driven every two weeks between 7:00 and 11:00hrs. Five transects followed the game viewing road along the northern bank of the GRR. Five transects followed game viewing roads leading away from the GRR in northerly directions.

### Mortality

Hippo carcasses were encountered and recorded during transects, censuses at monitoring locations and during incidental encounters, or were reported by National Park personnel and tourist guides and then verified.

### Statistical Analysis

The chi-square test of independence or association with the log likelihood ratio as test statistic was used to check whether the state of water at the monitoring locations was related to the topographic gradient [30].

We used generalised linear models (GLMs—here, binary and multinomial logistic, poisson and negative binomial regression models, see below) to assess the impact of predictor variables on the (1) change in the expanse of water (status) at monitoring locations, (2) minimum number of hippos at the 14 monitoring locations, (3) total number of hippos counted within the study area, and (4) chance of encountering females and calves in a group of hippos.

Potential predictors of the change in the expanse of water (status) at monitoring locations included the topographic gradient (steep versus flat), month as an index of the progression of dry season and year. We ran a multinomial logistic regression in Systat 13 to assess whether these predictors significantly affected the status and report the global summary of the effect of each predictor on the probability of occurrence of each status.

Potential predictors of the minimum number of hippos at 14 monitoring locations included the fixed effects of water quality (salinity and aerobic bacterial load), the state of the water at monitoring locations (scored in the four categories defined above), water flow (scored in the three categories defined above), the month, the year and the identity of the location. For monitoring locations which were not directly water-sampled, values of salinity, total aerobic bacteria load and water flow were assigned from the nearest water sampling point to them.

We selected a negative binomial regression model, after an initial fit with a fixed-effects Poisson regression model demonstrated substantial over-dispersion, since the residual scaled deviance was an order of magnitude larger than the residual degrees of freedom (e.g. [31]). The negative binomial regression model substantially reduced dispersion in this respect (dispersion parameter σ = 0.27). We fitted a mixed-effects zero-inflated negative binomial regression in order to account for the fact that counts were repeated at the same location and because a substantial number of counts observed an absence of hippos. The mixed-effects zero-inflated negative binomial regression was fitted by applying the function gamlssNP from package gamlss version 4.3–2 plus complement package gamlss.mx [32] in R [33]. We chose a nonparametric maximum likelihood approach in gamlssNP specifically designed to fit overdispersed generalized linear models and extended to generalized linear models with shared random effects which may originate from, for instance, a repeated measures structure [34, 35]. The census number for each dry season was entered as a random factor on the intercept, assuming a nonparametric discrete distribution of K intercept match points and thereby converting it essentially to a nonparametric model [31]. As recommended by [31], we varied the number of K intercept match points between K = 1 and K = 5 and chose the model with the lowest AIC (see below), which was the model with K = 1. We also calculated ρ, the proportion of the total variance explained by the random effect, as ρ = σ^2^ / (1 + σ^2^) where σ is the dispersion parameter [31]. Total sample size was n = 182 counts.

The total number of hippos counted within the study area during a census was analysed with a standard negative binomial regression model. Predictor variables included the number of monitoring locations censused and the year of observations. We checked for autocorrelation of residuals λ using function acf in R [32] to check for independence of data points. Lag 1 (immediate neighbour) data points showed a marginally significant level of λ of 0.433. In order to assess the consequences for testing the significance of predictor variables, we followed Cerioli’s approach [36], divided the difference between the log-likelihoods of the full and the reduced models (see below) by (1+λ) and then re-calculated the p-values. This correction had no effect on the significance of the p values for the predictor variables, so we are reasonably confident that the outcome of this model is sufficiently robust. The negative binomial regression was fitted by applying function glm.nb from package MASS version 7.3–43 [37] in R [33].

Potential predictors of the chance of encountering females, infants or juveniles in a group of hippos included the year (2012 or 2013), the stage of the dry season and the state of the water at monitoring locations. We ran a mixed-model binary logistic regression, with the census number for each monitoring location entered as a random factor on the intercept, for those data points when there was at least one hippo observed at a monitoring location (n = 86). This was therefore a subset of the data used to predict the minimum number of hippos at each monitoring location. Given the reduced sample size, we focused on predictors relating to the expanse of water at monitoring locations, the progression of the dry season (early, i.e. June to August, versus late, i.e. September to November) and the year of census (2012 versus 2013). The mixed-effects binary logistic regression was fitted by applying function glmer from package lme4 version 1.1–8 [38] in R [33].

We used log-likelihood ratio tests (G-tests) and information criteria (the Akaike Information Criterion [AIC], the quasi-likelihood Information Criterion [AIC_qh_] introduced by Hannan and Quinn [39] and Raftery’s Bayesian Information Criterion [BIC_R_]) to check whether the final model was superior to an intercept-only or a reduced model. Models were considered similar if differences in AIC were less than 2.5 and preferable if the difference exceeded 6.0 [40], similar if differences in BIC_R_ were less than 2.0, preferable if values of BIC_R_ varied between 2.01 and 6.0, and strongly preferable if values of BIC_R_ differed by more than 6 (A. Raftery in [31], p73). As the evaluation of our models with both Akaike and Bayesian information criteria produced similar conclusions, we report only AIC values. We also report the AIC_qh_ values, since they can be of interest in the case of substantial dispersion and were developed in the context of correlated data. The significance threshold of these tests was fixed at 5%. All tests were two-tailed, except for the test on the direction of movements of hippos encountered in transects, where because of the results on changes in the expanse of water at monitoring locations (Table 1) we expected hippos to be more likely to move upstream.

**Table 1:** Multinomial logistic regression of predictors affecting the chance of a change in the expanse of water at monitoring locations.

Predictor	Direction of effect on chance of expanse of water being in a given state*				df	G	p	AIC	ΔAIC	AIC_qh_	ΔAIC_qh_
	dry	shallow	moderate	large							
Year	-0.032	0.030	-0.118	0.120	3	11.593	0.0089	433.70	5.59	1.575	-0.029
	2012 < 2013	2012 > 2013	2012 < 2013	2012 > 2013							
Topographic gradient	-0.024	-0.120	-0.348	0.492	3	130.348	< 0.00001	552.45	124.35	1.986	0.382
	steep < flat	steep < flat	steep < flat	steep > flat							
Month of dry season	0.022	0.049	-0.035	-0.037	3	117.211	< 0.00001	539.31	111.21	1.941	0.336
	↑ as dry season progresses	↑ as dry season progresses	↓ as dry season progresses	↓ as dry season progresses							

Tests for significance of each parameter used log-likelihood ratio tests (G) with associated degrees of freedom (df) and p-values (p). Values for the Akaike Information Criterion (AIC) and the quasi-likelihood information criterion (AIC_qh_) and the respective differences to the full model (ΔAIC, ΔAIC_qh_) are shown for each alternative model when the specific predictor was removed. For the full model, AIC was 428.10 and AIC_qh_ was 1.605.

* Global change of the probability of each of the four states of the expanse of water in response to a change in the value of each predictor variable. The sum of the values for each predictor is 0, as an increase in the probability in one state must be compensated for by a decrease in other states.

The significance of each fixed-effects predictor variable was assessed as the marginal contribution of each parameter to the full model by subtracting from the full model the log-likelihood of a second model with each specific fixed effects predictor removed and testing the difference against a chi-square distribution with the appropriate degrees of freedoms (see discussions in [31, 41]).

Means are reported ± standard error of the mean. Statistical tests were performed using SPSS Statistics for Windows version 21.0 (IBM Corp, Armonk, NY; USA), Systat version 13 (Systat Software Inc., Richmond, VA, USA) and R version 3.2.2 [33].

The map in Fig 1 was created using Esri ArcGIS Desktop, ArcGIS release 10.3.1. (Environmental Systems Research Institute, Redlands, California, USA) and included a modified outline of the rivers derived from “Diva-GIS” [42] and a modified outline of the border of the National Park derived from “The World Database on Protected Areas” (WDPA) [43].

## Ethics Statement

The Tanzanian Commission of Science and Technology, the Tanzania Wildlife Research Institute approved the research and the Tanzania National Parks granted permission to conduct research in Ruaha National Park. The work was also approved by the Internal Ethics Committee of the Leibniz Institute for Zoo and Wildlife Research (IZW), Approval No. 2011-04-02.

## Results

The expanse of water (state) of monitoring locations was significantly associated with the topographic gradient, the progression of the dry season and significantly varied between years (multinomial logistic regression, log-likelihood ratio test, test statistic = 242.25, n = 289, p < 0.00001): locations along the upstream (south-westerly) section of the GRR with the steep topographic gradient were significantly more likely to be categorised as “large” in terms of the expanse of water than those along the downstream (north-easterly) section of the river with the flat gradient. As the dry season progressed, the chance of a monitoring location being in the states of “large” or “moderate” significantly decreased and being in the states of “shallow” or “dry” significantly increased. In 2013, monitoring locations were significantly less likely to be “large” and “shallow” and significantly more likely to be “moderate” and “dry”, suggesting that larger and smaller expanses of water shrank in a consistent manner from 2012 to 2013.

The mean minimum number of hippos counted (termed hereafter mean hippo counts) per monitoring location across both seasons was 10.5 ± 1.3 hippos (range 0 to 95, n = 207 counts), 13.3 ± 2.1 (range 0 to 95, n = 97 counts) in 2012 and 8.1 ± 1.6 hippos (range 0 to 80, n = 110 counts) in 2013.

### Changes in hippo distribution between the early and late dry season

**Fig 2:**
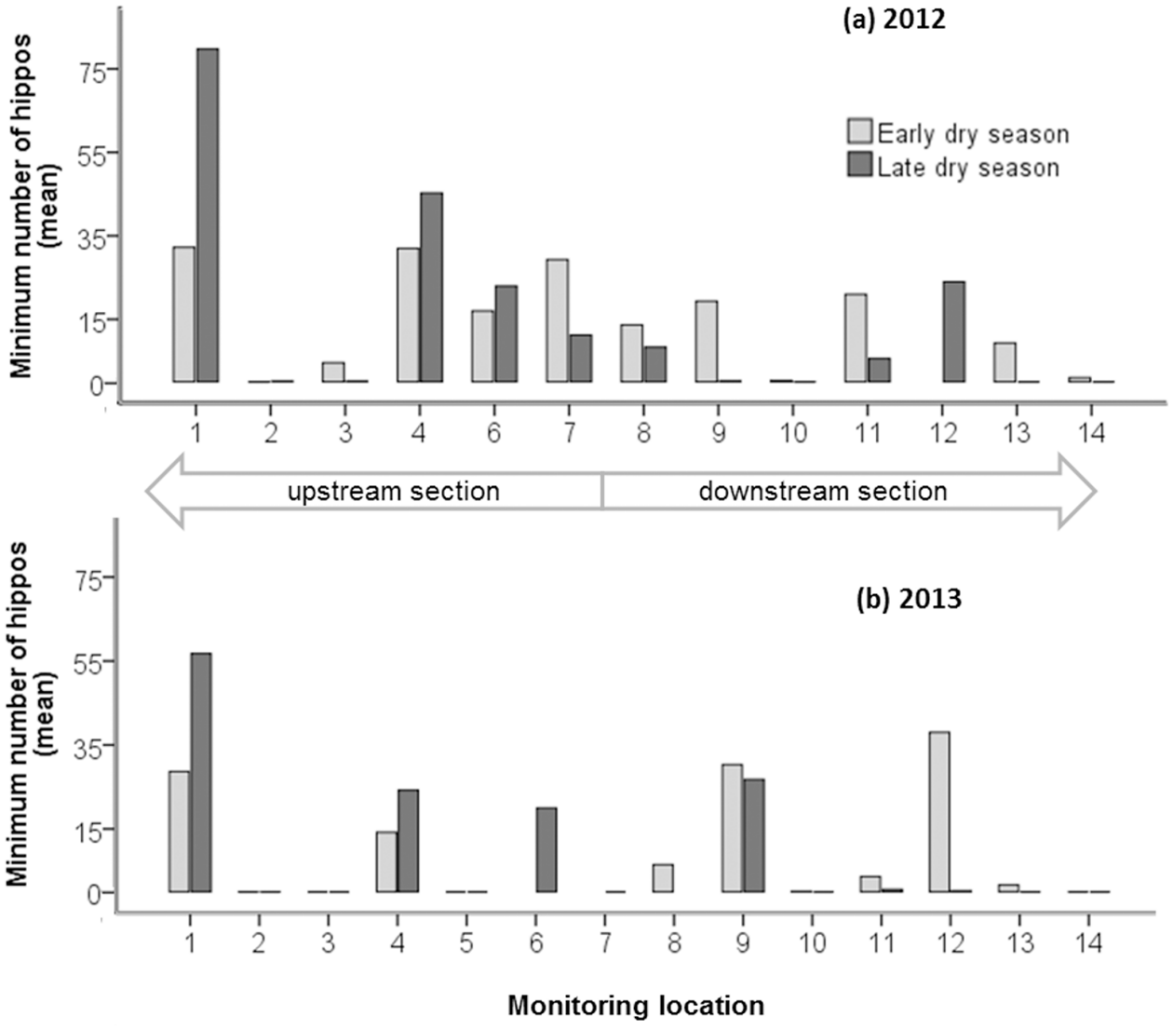
The mean minimum number of hippos at 14 monitoring locations along the Great Ruaha River, during the early dry season (June to August in light grey) and late dry season (September to November in dark grey) in (a) 2012 and (b) 2013. Locations 1–7 were defined as upstream and location 8–14 as downstream sections of the river. Thin black lines at zero on the x-axis indicate a mean minimum count of zero hippos at the location, an absence of a line or bar indicates that the location was not monitored.

Hippos occurred at more monitoring locations during the early than late dry season in both study years and the general overall trend was for hippos to move from downstream locations on the GRR to those upstream (Fig 2). The highest mean hippo counts were at locations 1 and 4 in the upstream section of the river in the late dry season period of 2012 and at location 1 in the late dry season of 2013. In 2013, both the early and late dry season mean hippo counts at location 4 were well below those at this location in 2012. In 2013, the early and late mean hippo counts at the downstream location 9 were similar, and both these counts were higher than those in 2012 (Fig 2). In 2013, the mean hippo count during the early dry season at downstream location 12 was relatively high but the mean late dry season count was only 0.25 animals, even though it was 24 animals in the late dry season of 2012.

**Fig 3:**
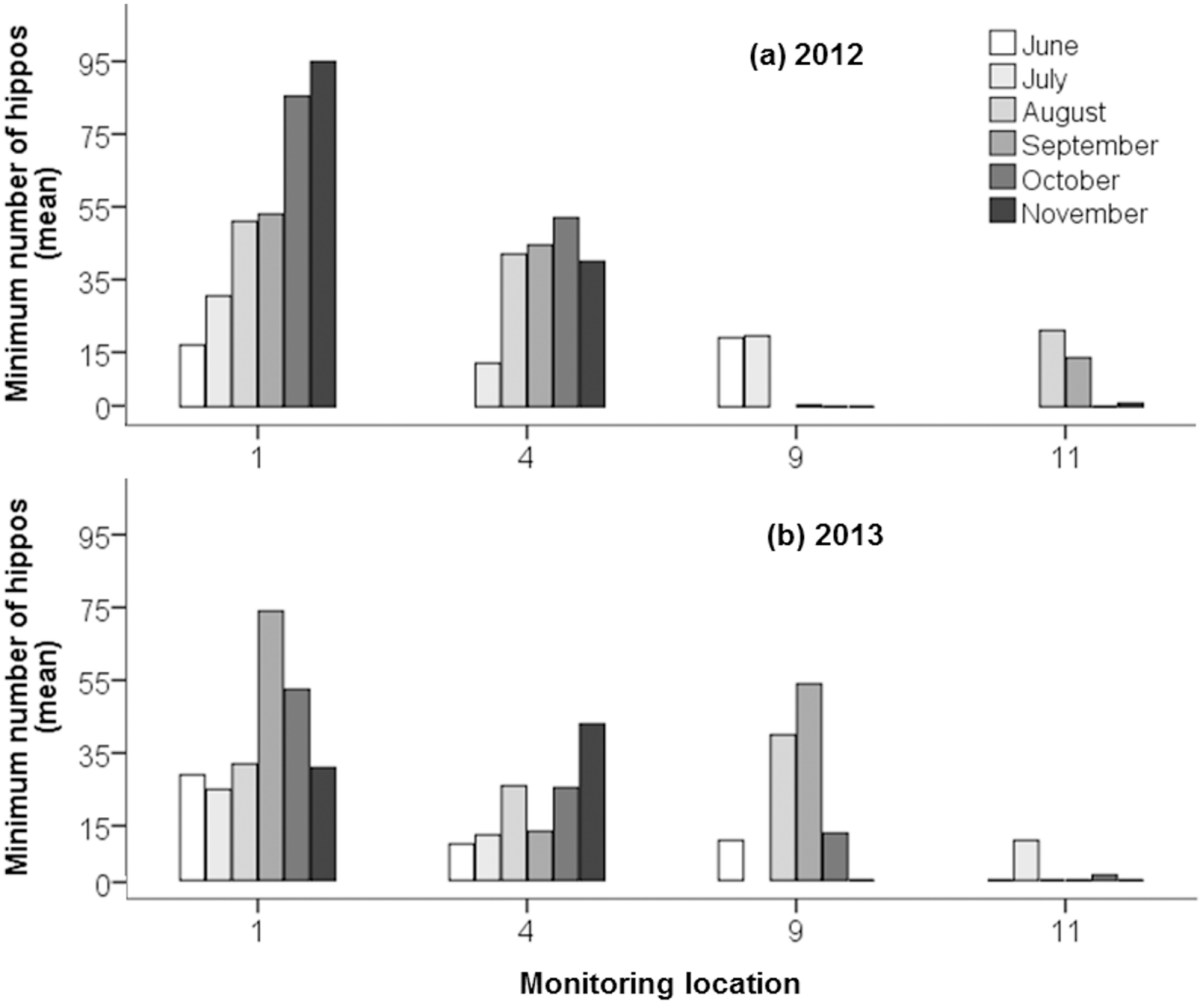
Mean minimum number (per month) of hippos during the (a) 2012 and (b) 2013 dry season (June to November) at monitoring locations 1 and 4 (upstream) and 9 and 11 (downstream) along the Great Ruaha River. A thin black line at zero on the x-axis indicates a count of zero hippos at the monitoring location, an absence of a line or bar indicates that no records were available from the monitoring location in that month.

A more detailed monthly examination (Fig 3) of changes in mean hippo counts at the two upstream locations 1 and 4 (Fig 1) and the two downstream locations 9 and 11 (Fig 1) revealed considerable changes in the mean hippo counts observed at these locations throughout the 2012 and 2013 dry seasons. At location 1, the mean hippo count was at its lowest (17 animals) in June 2012; counts increased throughout the dry season reaching the highest mean count (95 animals) in November. In contrast, at this location in 2013, the highest hippo count occurred in September (74 animals) and then declined. At location 4 in 2012, the mean hippo count increased in August and remained at roughly the same level until November. Between August and October mean hippo counts were generally lower in 2013 than in 2012. Apart from a single individual, all hippos had vacated location 9 by September 2012, whereas in 2013 mean hippo counts at this location in August and September were 40 animals and 54 animals respectively. No hippos were observed at location 9 in November in either dry season. Location 11 was typical of several downstream sites where a limited and changing number of hippos were counted in the early months of the dry season, but only single (presumably territorial bulls) remained there during the last months of the dry season.

### Factors influencing the number of hippos and the composition of hippo groups at monitoring locations

The minimum number of hippos per monitoring location significantly changed with its expanse of water (status), significantly differed between the 14 monitoring locations and significantly increased with the progression of the dry season (mixed-model zero-inflated negative binomial regression, log-likelihood ratio test = 201.6, df = 20, p < 0.00001, Table 2, n = 182, Table 2). Salinity, total aerobic bacterial load, water flow and year had no influence on the minimum number of hippos counted at monitoring locations (Table 2). The proportion ρ of 0.068, the contribution of the random effect (census number), explained a modest amount of the total variance.

**Table 2:** Mixed-model zero-inflated negative binomial regression of predictors affecting the minimum number of hippos per location.

Predictor	Estimate	SE	df	G	p	AIC	ΔAIC	AIC_qh_	ΔAIC_qh_
Intercept	700	396.3		1.766^a^	0.079	916.59^b^	158.82	5.039^b^	0.301
Census number (random effect, intercept)	0.144	0.195		0.736^a^^,^^b^	0.46				
State of water at location: shallow < moderate ≈ large									
shallow	-4.002	-4.002	2	29.959	<0.00001	783.73	25.96	4.812	0.074
moderate	-0.459	-0.459							
Water flow: (flowing ≥ stagnant)	0.320	0.400	1	0.741	0.39	756.51	-1.26	4.696	-0.042
Salinity	0.003	0.002	1	3.016	0.082	758.78	1.02	4.709	-0.029
Total aerobic bacterial load	-0.00008	0.0005	1	0.054	0.82	755.82	-1.95	4.693	-0.045
Month of dry season (linear trend)	0.270	0.150	1	4.134	0.042	759.90	2.13	4.715	-0.023
Square of the month of dry season (quadratic trend)	-0.024	0.013	1	4.076	0.043	759.84	2.07	4.715	-0.023
Year: (2013 ≤ 2012)	-0.347	0.197	1	2.406	0.12	758.17	0.40	4.706	-0.032
Monitoring location along the river (see Fig 1)									
1* (steep)	1.130	0.520	13	134.1	<0.00001	865.88	108.11	4.927	0.189
2* (steep)	-5.225	1.119							
3* (steep)	-1.723	0.488							
4	0.264	0.508							
5* (steep)	-2.6 * 10^16^	2.1 * 10^15^							
6	-0.088	1.128							
7	0.159	0.496							
9	0.604	0.321							
10* (flat)	-3.523	0.856							
11	0.562	0.684							
12	0.827	0.596							
13	-0.536	0.664							
14* (flat)	-3.678	1.091							

Shown are regression coefficients (estimates) and standard errors (SE) of predictors in natural log-units (logits). Positive (negative) estimates indicate that an increase in the value of the predictor increased (reduced) the minimum number of hippos. Tests for significance of each parameter used log-likelihood ratio tests (G) with associated degrees of freedom (df) and p-values (p). Values for the Akaike Information Criterion (AIC) and the quasi-likelihood information criterion (AIC_qh_) and the respective differences to the full model (ΔAIC, ΔAIC_qh_) for each reduced model are shown when the specific predictor was removed. For the full model, AIC was 757.77 and AIC_qh_ was 4.738.

^a^ based on Wald tests (z-values = estimate/SE and their associated p-values);

^b^ intercept-only model

* significantly different from the reference monitoring location (“8”, Fig 1)

The likelihood of groups of hippos containing infants, juveniles and/or females significantly increased with the state of the water expanse per monitoring location, improving from “shallow” via “medium” to “large” (mixed-model binary logistic regression, log-likelihood ratio test = 16.691, df = 4, n = 86, p = 0.0022, Table 3), and was independent of the year and the stage of the dry season (Table 3).

**Table 3:** Mixed-model binary logistic regression of predictors affecting the likelihood of encountering females and calves per monitoring location.

Predictor	Estimate	SE	df	G	p	AIC	ΔAIC	AIC_qh_	ΔAIC_qh_
Intercept	-1.230	1.346		-0.914^a^	0.36	73.51^b^	8.69	0.840	-0.024
Census number (random effect, intercept)	1.819	0.311		5.846^a^^,^^b^	<0.00001				
State of water at location: large > moderate ≥ shallow									
Moderate	2.706	1.521	2	14.144	0.00085	74.96	10.14	0.908	0.043
Large	4.916	1.699							
Year: (2013 > 2012)	1.421	0.895	1	3.048	0.081	65.87	1.05	0.836	-0.027
Season: (early [Jun to August] ≤ late [September to November])	-1.056	0.882	1	1.623	0.20	64.44	-0.38	0.820	-0.044

Shown are the regression coefficients (estimates) and their standard errors (SE) of predictors in natural log-units (logits). Positive (negative) estimates indicate that an increase in the value of the predictor increased (reduced) the likelihood of encountering females and calves per monitoring location. Tests for significance of each parameter used log-likelihood ratio tests (G) with associated degrees of freedom (df) and p-values (p). Values for the Akaike Information Criterion (AIC) and the quasi-likelihood information criterion (AIC_qh_) and the respective differences to the full model (ΔAIC, ΔAIC_qh_) are shown for each alternative model when the specific predictor was removed. For the full model, AIC was 64.82, AIC_qh_ was 0.864.

^a^ based on Wald tests (z-values = estimate/SE and their associated p-values);

^b^ intercept-only model

### Camera trap records

We obtained 78 and 83 independent hippo camera trapping events in 2012 and 2013, respectively. Hippos were captured at most (6 of 7) camera trap positions along the GRR and only few (2 of 7) camera traps positioned at water sources away from the GRR (non-GRR positions, Table 4). No hippo cows or immature hippos were detected by camera traps at the furthest downstream camera traps (G and F) on the GRR from September to November in both study years (Table 4). Hippo cows with infants and/or juveniles were recorded by camera trap positions A, B, D and E throughout both dry seasons. At position C a single hippo bull was detected in September 2012. Camera traps at non-GRR positions only captured solitary males. The two non-GRR positions furthest away from the GRR (L and N, Fig 1) were located at a distance of 3 km and 5.7 km from the GRR.

**Table 4:** Independent camera trapping events of hippos at monitoring locations during the dry season in 2012 and 2013.

Year	2012						2013					
Month	JUN	JUL	AUG	SEP	OCT	NOV	JUN	JUL	AUG	SEP	OCT	NOV
Cameratrap position (site)												
A (GRR)	-	-	-	2,3,1,4	1	2,3,1,4	0	2,3	0	2,1,3	2,3	-
B (GRR)	-	-	-	2,4	0	2,3,	0	2,1,3,4	0	0	0	-
C (GRR)	-	-	-	1	0	2,	0	0	0	0	0	-
D (GRR)	-	-	-	0	0	2,1,	4	0	0	0	2,3	-
E (GRR)	-	-	-	1	2,1	2,3,4	2,1,4,	2,3	2,1,3	2,1,4,	2,1	-
F (GRR)	-	-	-	0	0	0	1,	0	0	0	0	-
G (GRR)	-	-	-	0	1	2,1	2,4	0	0	0	0	-
H (Non-GRR)	-	-	0	0	0	-	-	0	0	-	0	0
I (Non-GRR)	-	-	0	0	0	-	-	0	0	-	0	0
J (Non-GRR)	-	-	0	0	0	-	-	0	0	-	0	0
K (Non-GRR)	-	-	0	0	0	-	-	0	0	-	0	0
L (Non-GRR)	-	-	0	0	0	-	-	1	1	-	0	1
M (Non-GRR)	-	-	0	0	0	-	-	0	0	-	0	0
N (Non-GRR)	-	-	1	0	0	-	-	1	0	-	0	0

Numbers indicate the sex and number of animals captured if a camera trap was activated (dash: no camera trap activated): 0 = 0 hippos; 1 = single adult male; 2 = single adult female or single adult unknown; 3 = at least one immature with one or more adults; 4 = more than one adult. GRR: location at Great Ruaha River; Non-GRR: location at water sources away from the river

### Transects

Twenty five hippos were recorded on land during daytime transects (Table 5). All were sighted along the GRR either as solitary animals or in small groups, with a mean group size of 4.4 ± 1.9. All groups for which a direction of movements could be clearly identified were walking upstream in the dry river bed, a result unlikely to be a consequence of chance alone (Wilcoxon signed-ranks test, W = 15.0, exact p = 0.031, one-tailed). Such daytime movements of hippos were not observed in the early dry season in June. One larger group of 12 animals was observed moving upstream in October 2012. Hippos were not seen on any transects leading away from the GRR.

**Table 5:** Observations of hippos on land during transects along the Great Ruaha River.

Site (closest monitoring location)	Date	Time	Number of individuals	Adults	Juveniles	Special observation
3	06 Jun 2012	8:35	2	2	0	-
11	12 Sep 2012	8:28	1	1	0	walk upstream
13	12 Oct 2012	8:55	12	9	3	walk upstream
12	12 Oct 2012	9:06	3	2	1	walk upstream
11	15 Jul 2013	8:20	2	2	0	walk upstream
8	05 Aug 2013	7:02	1	1	0	-
2	12 Aug 2013	8:36	1	1	0	-
13	30 Oct 2013	8:29	3	2	1	walk upstream

### Mortality

Of nine cases of observed mortality, six occurred in 2012 and three in 2013. Mortality in three adult hippo bulls on 15th September 2012, 7th October 2012 and 8th August 2013 were attributed to intraspecific aggression based on the nature of the severe wounds on the carcasses or observed intense intraspecific aggression. Three juveniles died without signs of external wounds on 27th August 2012, 1st November 2012 and 21st August 2013, respectively. Lion predation of one calf was recorded on 28th August 2012, one adult most likely died from malnutrition on 25th September 2012, and one adult died of unknown causes on 23rd July 2013.

### Minimum population size of hippos

**Fig 4:**
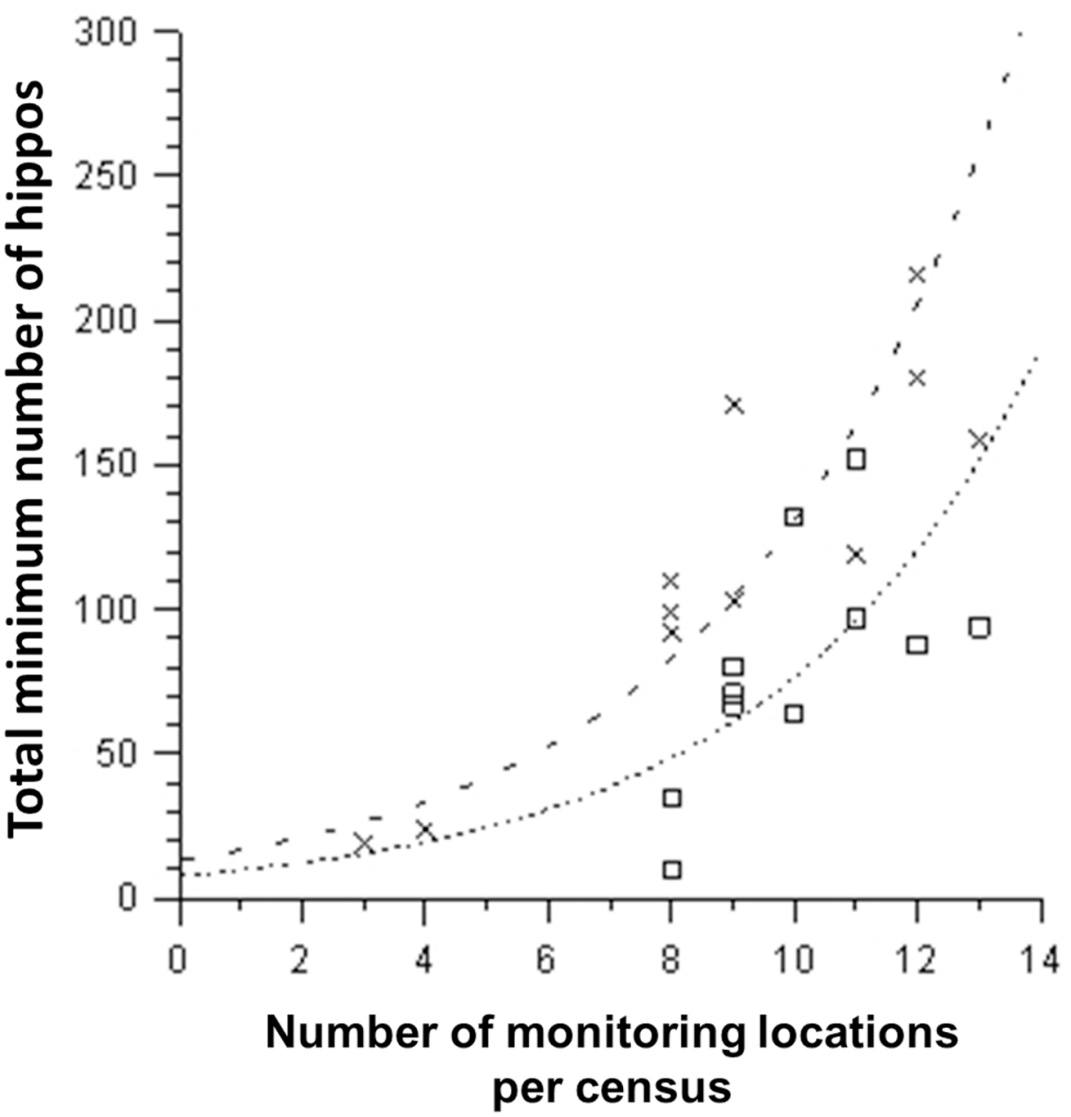
Minimum total numbers of hippos along the Great Ruaha River as a function of the number of hippo pools counted during the dry season 2012 and the dry season of 2013. Dashed (for 2012 crosses) and dotted (for 2013 squares) are the regression lines from the negative binomial model.

If we assume that the distribution of hippos in the 14 monitoring locations did not change substantially between counts on different days along the three censusing sections of the GRR during each census, then the sum of the minimum numbers counted per location across these sections provides an approximate estimate of the hippo population along the monitored section of the GRR (Fig 1). The highest number of hippos observed during a census was 216 in 2012 and 152 in 2013. The total minimum number of hippos per census significantly increased (negative binomial regression, overall model, log-likelihood ratio test, test statistic = 22.167, df = 2, p = 0.000015, p adjusted for autocorrelation = 0.00044, Fig 4) with the number of monitoring locations at which hippos were counted during any one census (log-likelihood ratio test, test statistic = 20.115, df = 1 p = 0.0000073, p adjusted for autocorrelation = 0.00018) and was significantly higher during 2012 than 2013 (log-likelihood ratio test, test statistic = 8.170, df = 1 p = 0.0043, p adjusted for autocorrelation = 0.017).

## Discussion

Our results revealed that, in both study years, an estimated population of between 152 and 216 hippos were more dispersed during the early than the late dry season (Fig 2) along the GRR in the Ruaha NP (Fig 1). As the dry season progressed, the incidence of water flow and the availability of surface water in the GRR declined (Table 1), hippos abandoned unsuitable daytime resting locations and moved to other, more suitable ones (Table 2, Fig 3). This resulted in the congregation of hippos in relatively large numbers in a few locations (Fig 2), with the highest mean counts recorded at location 1 at the end of the dry season in November 2012 (Fig 3), at the upstream end of the study area (Fig 1). The results of our model (Table 2) revealed that minimum hippo numbers per monitoring location on the GRR increased with the progression of the dry season and were significantly higher as the expanse of available surface water per monitoring location increased. Groups of hippos were more likely to contain infants, juveniles and/or females, regardless of the year or stage of the dry season, as the expanse of water per monitoring location increased (Table 3). Consistent with the finding of studies on other hippo populations in Africa [8, 17, 44, 45] our results indicate that when water dries up at a resting location, hippos move to a more suitable one. The availability of suitable daytime resting locations along the GRR declined during the dry season, particularly in the downstream section, causing hippos to congregate in a relatively limited number of locations, the majority of which were located in the upstream section of the river (Fig 2). During both dry seasons, the downstream section of the GRR contained extensive sections of dry riverbed or relatively small isolated pools of water [25] which, if not empty, were mostly occupied by solitary male hippos (Table 4). We expected hippos to prefer locations with better water quality, in terms of water flow, lower salinity and total aerobic bacterial load, but found no evidence for this (Table 2).

There were significant differences between minimum hippo numbers per monitoring location in 2012 and 2013 (Table 2). We speculate that this difference between years may be explained by more hippos in 2013 moving to locations further upstream of location 1 (Fig 1) and hence outside our study area than in 2012. In 2012, the largest aggregation of hippos was recorded at location 1 at the end of the dry season in October and November (Fig 3), whereas in 2013, the peak count at this location occurred in September, and then counts declined in both October and November, suggesting movement of hippos further upstream. This idea is consistent with more monitoring locations in 2013 having few or no hippos than in 2012 (Fig 2), which suggests that during the 2013 dry season more daytime resting locations were unsuitable for moderate to large herds of hippos than in 2012, and hence were responsible for the smaller estimated total number of hippos in the study area in 2013 than 2012 (Fig 4). Our sightings of hippo groups only walking upstream (Table 5) during daylight hours is also consistent with our suggestion that hippos searching for daytime resting sites predominantly move upstream.

We present several lines of evidence (hippo censuses, transect data, camera trap data) that the vast majority of hippos in our large study area depended on the GRR for their day resting locations, and that location 1 was important for a large number of animals during the late dry season. Our camera trapping data revealed that only a few solitary males occurred at water sources away from the GRR during the dry season.

Our limited results on hippo mortality are consistent with our expectation that intra-specific aggression is a mortality factor during the dry season. Although 2013 was apparently a less favourable year for hippos than 2012, we detected fewer cases of mortality in 2013 than 2012. We suggest that this may be due to fewer hippos remaining in our study area throughout the late dry season of 2013 because many probably moved upstream and beyond our study area, hence reducing our chance of observing mortality. Hippos in large aggregations and those forced to move during daylight hours to find daytime resting sites are likely to experience elevated levels of physiological stress [17, 19, 46] and the possible negative consequences this might have on immune processes may be compounded by increased feeding competition at night, particularly in females that have to increase nutrient intake to fulfil the high nutritional demands of pregnancy and lactation [47–49]. Although disease transmission can be facilitated by high host densities we found no evidence of this during our relatively short-term study. We observed three incidences of hippos mating in the GRR in June and July (personal obs), which is also consistent with the idea that oestrus can occur during the dry season and hence oestrus females forced to use daytime resting locations outside their normal range may be particularly vulnerable to sexual harassment.

We speculate that the scale of changes in hippo daytime resting locations revealed by our study is likely to be more extreme, in terms of the numbers of relocations undertaken by hippos and the distances travelled to alternative locations, than those before the significant reduction in the GRR dry season flow since 1993, attributed to large-scale water extraction for agriculture, upstream of the Ruaha NP [24]. Females with wet season ranges in the furthest downstream section of our study area are likely to have relocated by at least 80 km by the end of the dry season. Females and their offspring probably move upstream from one localised source of water to another, until they eventually reach a more permanent site such as location 1. Even so, in some years, may have to move even further upstream, as indicated by our results from 2013.

Are these changes likely to have negative consequences for the hippo population? To what extent is the resilience and long-term prospect of the hippo population compromised if human water extraction continues at current levels, or increases with human population growth? As in so many conservation issues, data prior to the start of this anthropogenic change are, to our knowledge, not available, hence assessing the scale of its impact is problematic. We approach this issue by considering some evidence that allows us to estimate what proportion of the entire hippo population of the Ruaha ecosystem (approximately 45,000 km^2^) resides in Ruaha NP, and in our study area, and look at the results of previous aerial transect surveys of the wildlife populations of the Greater Ruaha ecosystem. Previous ‘counts’ of hippos in the Greater Ruaha ecosystem were a by-product of dry season aerial transect surveys of large mammals conducted in 1993, 1999, 2002, 2006 and 2009 [27, 28]. Although these surveys were not at all suited to provide reliable quantitative estimates of population size (and thus are not really suitable to indicate population trends), they provide at least an index of the distribution of the hippo population. They indicate that during the dry season more than 90% of the entire hippo population of the Greater Ruaha ecosystem appeared to be restricted to the GRR inside the National Park (e.g. the dry season survey in October 2009, [28]).

One of the main threats to hippos is habitat loss caused by humans (e.g. [5–7]). The results of our study indicate that human extraction of water from the GRR is changing the distribution of the hippo population within Ruaha NP. As a considerable part of the GRR within our study area dried out towards the end of dry season [25], this represented an extensive loss of dry season habitat for the hippo population in Ruaha NP. The resilience of the hippo population to these changes is currently unknown, but requires investigation.

## Supporting Information

S1 Table Supporting information file, including data used for the mixed-model negative binomial regression. Data ordered by Year (2012, 2013), Month (in two week intervals, first half of the month and second half of the month from June to November) and Monitoring location (1–14). Other variables are Census number for each dry season (1–11), categorization of Season (early/late), the classification of locations in terms of topography (steep/flat), Salinity, total aerobic bacterial load, Water flow (1–3), State of Water at monitoring location (1–4), Presence of females, infants and/or juveniles and the minimum number of hippos present.
(PDF)
