# Supplementary material for: The Effect of Reduced Water Availability in the Great Ruaha River on the Vulnerable Common Hippopotamus in the Ruaha National Park, Tanzania
Source: PLoS One. 2016 Jun 8;11(6):e0157145. doi: 10.1371/journal.pone.0157145 (PMC4898818; doi:10.1371/journal.pone.0157145)
Supplement: S1 Table — Data ordered by Year (2012, 2013), Month (in two week intervals, first half of the month and second half of the month from June to November) and Monitoring location (1–14). Other variables are Census number for each dry season (1–11), categorization of Season (early/late), the classification of locations in terms of topography (steep/flat), Salinity, total aerobic bacterial load, Water flow (1–3), State of Water at monitoring location (1–4), Presence of females, infants and/or juveniles and the minimum number of hippos present. (PDF) [file pone.0157145.s001.pdf]

Year	Month	Census number	Season	Monitoring Location	Steepflat	Salinity	Total_aerobic_bacterial_load	Flow	State_of_water_location	Females, infants, or juveniles_present	Min_num_hippos
2012	June I	1	early	1	steep	151	720	3_flowing	4_large_deep	1	17
2012	June I	1	early	2	steep	148	765	3_flowing	4_large_deep	NA	0
2012	June I	1	early	3	steep	160	775	3_flowing	4_large_deep	0	7
2012	June I	1	early	4	steep	157	675	3_flowing	4_large_deep	NA	NA
2012	June I	1	early	5	steep	143	660	3_flowing	3_small_deep	NA	NA
2012	June I	1	early	6	steep	143	660	3_flowing	4_large_deep	NA	NA
2012	June I	1	early	7	steep	143	660	3_flowing	4_large_deep	NA	NA
2012	June I	1	early	8	flat	144	590	3_flowing	3_small_deep	NA	NA
2012	June I	1	early	9	flat	144	590	3_flowing	4_large_deep	NA	NA
2012	June I	1	early	10	flat	144	590	3_flowing	3_small_deep	NA	0
2012	June I	1	early	11	flat	156	605	3_flowing	3_small_deep	NA	NA
2012	June I	1	early	12	flat	156	605	3_flowing	3_small_deep	NA	NA
2012	June I	1	early	13	flat	156	605	3_flowing	4_large_deep	NA	NA
2012	June I	1	early	14	flat	156	605	3_flowing	3_small_deep	NA	NA
2012	June II	2	early	1	steep	152	730	3_flowing	4_large_deep	NA	NA
2012	June II	2	early	2	steep	158	750	3_flowing	4_large_deep	NA	0
2012	June II	2	early	3	steep	171	710	3_flowing	4_large_deep	NA	NA
2012	June II	2	early	4	steep	134	750	3_flowing	4_large_deep	NA	NA
2012	June II	2	early	5	steep	140	595	3_flowing	3_small_deep	NA	NA
2012	June II	2	early	6	steep	140	595	3_flowing	4_large_deep	NA	NA
2012	June II	2	early	7	steep	140	595	3_flowing	4_large_deep	NA	NA
2012	June II	2	early	8	flat	145	605	3_flowing	3_small_deep	NA	NA
2012	June II	2	early	9	flat	145	605	3_flowing	4_large_deep	1	19
2012	June II	2	early	10	flat	145	605	3_flowing	3_small_deep	NA	0
2012	June II	2	early	11	flat	172	675	3_flowing	3_small_deep	NA	NA
2012	June II	2	early	12	flat	172	675	3_flowing	3_small_deep	NA	NA
2012	June II	2	early	13	flat	172	675	3_flowing	4_large_deep	NA	NA
2012	June II	2	early	14	flat	172	675	3_flowing	3_small_deep	NA	NA
2012	July I	3	early	1	steep	148	575	3_flowing	4_large_deep	1	25

2012	July I	3	early	2	steep	149	655	3_flowing	4_large_deep	NA	0
2012	July I	3	early	3	steep	173	690	3_flowing	4_large_deep	1	5
2012	July I	3	early	4	steep	151	690	3_flowing	4_large_deep	1	12
2012	July I	3	early	5	steep	154	650	3_flowing	3_small_deep	NA	NA
2012	July I	3	early	6	steep	154	650	3_flowing	4_large_deep	NA	NA
2012	July I	3	early	7	steep	154	650	3_flowing	4_large_deep	1	22
2012	July I	3	early	8	flat	159	645	3_flowing	3_small_deep	1	16
2012	July I	3	early	9	flat	159	645	3_flowing	4_large_deep	1	22
2012	July I	3	early	10	flat	159	645	3_flowing	3_small_deep	NA	0
2012	July I	3	early	11	flat	195	540	3_flowing	3_small_deep	NA	NA
2012	July I	3	early	12	flat	195	540	3_flowing	3_small_deep	NA	NA
2012	July I	3	early	13	flat	195	540	3_flowing	4_large_deep	NA	NA
2012	July I	3	early	14	flat	195	540	3_flowing	3_small_deep	0	1
2012	July II	4	early	1	steep	141	560	3_flowing	4_large_deep	1	36
2012	July II	4	early	2	steep	137	550	3_flowing	4_large_deep	NA	0
2012	July II	4	early	3	steep	155	585	3_flowing	4_large_deep	1	7
2012	July II	4	early	4	steep	153	660	3_flowing	4_large_deep	NA	NA
2012	July II	4	early	5	steep	152	570	3_flowing	3_small_deep	NA	NA
2012	July II	4	early	6	steep	152	570	3_flowing	4_large_deep	NA	NA
2012	July II	4	early	7	steep	152	570	3_flowing	4_large_deep	1	21
2012	July II	4	early	8	flat	170	520	3_flowing	3_small_deep	1	14
2012	July II	4	early	9	flat	170	520	3_flowing	4_large_deep	1	17
2012	July II	4	early	10	flat	170	520	3_flowing	3_small_deep	0	1
2012	July II	4	early	11	flat	176	560	3_flowing	3_small_deep	NA	NA
2012	July II	4	early	12	flat	176	560	3_flowing	3_small_deep	NA	NA
2012	July II	4	early	13	flat	176	560	3_flowing	4_large_deep	1	14
2012	July II	4	early	14	flat	176	560	3_flowing	3_small_deep	NA	NA
2012	Aug I	5	early	1	steep	175	685	3_flowing	4_large_deep	NA	NA
2012	Aug I	5	early	2	steep	175	760	3_flowing	3_small_deep	NA	0
2012	Aug I	5	early	3	steep	231	685	3_flowing	4_large_deep	1	4
2012	Aug I	5	early	4	steep	171	615	3_flowing	4_large_deep	1	48
2012	Aug I	5	early	5	steep	161	525	3_flowing	3_small_deep	NA	NA
2012	Aug I	5	early	6	steep	161	525	3_flowing	4_large_deep	1	17

2012	Aug I	5	early	7	steep	161	525	3_flowing	4_large_deep	NA	NA
2012	Aug I	5	early	8	flat	188	565	3_flowing	3_small_deep	NA	NA
2012	Aug I	5	early	9	flat	188	565	3_flowing	3_small_deep	NA	NA
2012	Aug I	5	early	10	flat	188	565	3_flowing	3_small_deep	NA	0
2012	Aug I	5	early	11	flat	203	640	3_flowing	3_small_deep	1	15
2012	Aug I	5	early	12	flat	203	640	3_flowing	3_small_deep	NA	NA
2012	Aug I	5	early	13	flat	203	640	3_flowing	3_small_deep	1	14
2012	Aug I	5	early	14	flat	203	640	3_flowing	3_small_deep	0	1
2012	Aug II	6	early	1	steep	156	900	3_flowing	4_large_deep	1	51
2012	Aug II	6	early	2	steep	181	855	3_flowing	3_small_deep	NA	0
2012	Aug II	6	early	3	steep	185	775	3_flowing	4_large_deep	NA	0
2012	Aug II	6	early	4	steep	205	735	3_flowing	4_large_deep	1	36
2012	Aug II	6	early	5	steep	170	600	3_flowing	3_small_deep	NA	NA
2012	Aug II	6	early	6	steep	170	600	3_flowing	4_large_deep	NA	NA
2012	Aug II	6	early	7	steep	170	600	3_flowing	4_large_deep	1	45
2012	Aug II	6	early	8	flat	234	780	3_flowing	3_small_deep	1	11
2012	Aug II	6	early	9	flat	234	780	3_flowing	3_small_deep	NA	NA
2012	Aug II	6	early	10	flat	234	780	3_flowing	3_small_deep	0	1
2012	Aug II	6	early	11	flat	218	825	3_flowing	3_small_deep	1	27
2012	Aug II	6	early	12	flat	218	825	3_flowing	3_small_deep	NA	NA
2012	Aug II	6	early	13	flat	218	825	3_flowing	3_small_deep	NA	0
2012	Aug II	6	early	14	flat	218	825	3_flowing	2_shallow	NA	NA
2012	Sep I	7	early	1	steep	165	865	3_flowing	4_large_deep	1	53
2012	Sep I	7	early	2	steep	182	705	3_flowing	3_small_deep	0	1
2012	Sep I	7	early	3	steep	175	780	3_flowing	4_large_deep	0	1
2012	Sep I	7	early	4	steep	190	740	3_flowing	4_large_deep	1	43
2012	Sep I	7	early	5	steep	188	745	3_flowing	2_shallow	NA	NA
2012	Sep I	7	early	6	steep	188	745	3_flowing	4_large_deep	1	24
2012	Sep I	7	early	7	steep	188	745	3_flowing	4_large_deep	1	33
2012	Sep I	7	early	8	flat	250	700	3_flowing	3_small_deep	NA	NA
2012	Sep I	7	early	9	flat	250	700	3_flowing	3_small_deep	NA	0
2012	Sep I	7	early	10	flat	250	700	3_flowing	2_shallow	NA	0
2012	Sep I	7	early	11	flat	266	1045	3_flowing	3_small_deep	1	27

2012	Sep I	7	early	12	flat	266	1045	3_flowing	3_small_deep	1	34
2012	Sep I	7	early	13	flat	266	1045	3_flowing	3_small_deep	NA	0
2012	Sep I	7	early	14	flat	266	1045	3_flowing	2_shallow	NA	0
2012	Sep II	8	late	1	steep	182	875	3_flowing	4_large_deep	NA	NA
2012	Sep II	8	late	2	steep	203	705	3_flowing	3_small_deep	NA	0
2012	Sep II	8	late	3	steep	207	725	3_flowing	4_large_deep	NA	0
2012	Sep II	8	late	4	steep	208	625	3_flowing	4_large_deep	1	46
2012	Sep II	8	late	5	steep	218	625	3_flowing	2_shallow	NA	NA
2012	Sep II	8	late	6	steep	218	625	3_flowing	4_large_deep	1	26
2012	Sep II	8	late	7	steep	218	625	3_flowing	4_large_deep	NA	NA
2012	Sep II	8	late	8	flat	338	685	3_flowing	3_small_deep	1	6
2012	Sep II	8	late	9	flat	338	685	3_flowing	3_small_deep	0	1
2012	Sep II	8	late	10	flat	338	685	3_flowing	2_shallow	NA	0
2012	Sep II	8	late	11	flat	335	770	3_flowing	2_shallow	NA	0
2012	Sep II	8	late	12	flat	335	770	3_flowing	3_small_deep	1	40
2012	Sep II	8	late	13	flat	335	770	3_flowing	3_small_deep	NA	0
2012	Sep II	8	late	14	flat	335	770	3_flowing	2_shallow	NA	0
2012	Oct I	9	late	1	steep	242	745	3_flowing	4_large_deep	1	81
2012	Oct I	9	late	2	steep	241	710	3_flowing	3_small_deep	NA	0
2012	Oct I	9	late	3	steep	263	705	3_flowing	2_shallow	NA	0
2012	Oct I	9	late	4	steep	274	1045	3_flowing	4_large_deep	1	52
2012	Oct I	9	late	5	steep	306	810	2_stagnant	2_shallow	NA	NA
2012	Oct I	9	late	6	steep	306	810	2_stagnant	4_large_deep	NA	NA
2012	Oct I	9	late	7	steep	306	810	2_stagnant	3_small_deep	1	9
2012	Oct I	9	late	8	flat	338	710	2_stagnant	3_small_deep	1	16
2012	Oct I	9	late	9	flat	338	710	2_stagnant	2_shallow	NA	0
2012	Oct I	9	late	10	flat	338	710	2_stagnant	2_shallow	NA	0
2012	Oct I	9	late	11	flat	398	2500	2_stagnant	2_shallow	NA	0
2012	Oct I	9	late	12	flat	398	2500	2_stagnant	3_small_deep	1	22
2012	Oct I	9	late	13	flat	398	2500	2_stagnant	3_small_deep	NA	0
2012	Oct I	9	late	14	flat	398	2500	2_stagnant	2_shallow	NA	0
2012	Oct II	10	late	1	steep	276	1300	3_flowing	4_large_deep	1	90
2012	Oct II	10	late	2	steep	313	815	3_flowing	3_small_deep	NA	0

2012	Oct II	10	late	3	steep	360	1040	2_stagnant	2_shallow	NA	0
2012	Oct II	10	late	4	steep	452	1775	2_stagnant	4_large_deep	NA	NA
2012	Oct II	10	late	5	steep	308	980	2_stagnant	2_shallow	NA	NA
2012	Oct II	10	late	6	steep	308	980	2_stagnant	4_large_deep	NA	NA
2012	Oct II	10	late	7	steep	308	980	2_stagnant	3_small_deep	1	2
2012	Oct II	10	late	8	flat	NA	NA	3_flowing	3_small_deep	NA	NA
2012	Oct II	10	late	9	flat	NA	NA	3_flowing	2_shallow	NA	NA
2012	Oct II	10	late	10	flat	NA	NA	3_flowing	2_shallow	NA	0
2012	Oct II	10	late	11	flat	338	1595	2_stagnant	2_shallow	NA	0
2012	Oct II	10	late	12	flat	338	1595	2_stagnant	NA	NA	NA
2012	Oct II	10	late	13	flat	338	1595	2_stagnant	2_shallow	NA	0
2012	Oct II	10	late	14	flat	338	1595	2_stagnant	1_dry	NA	0
2012	Nov I	11	late	1	steep	373	1130	2_stagnant	4_large_deep	1	95
2012	Nov I	11	late	2	steep	421	1090	2_stagnant	3_small_deep	NA	0
2012	Nov I	11	late	3	steep	467	590	2_stagnant	2_shallow	NA	0
2012	Nov I	11	late	4	steep	450	NA	2_stagnant	4_large_deep	1	40
2012	Nov I	11	late	5	steep	NA	NA	1_dry	2_shallow	NA	NA
2012	Nov I	11	late	6	steep	NA	NA	1_dry	4_large_deep	1	19
2012	Nov I	11	late	7	steep	NA	NA	1_dry	2_shallow	0	1
2012	Nov I	11	late	8	flat	275	1460	3_flowing	3_small_deep	1	3
2012	Nov I	11	late	9	flat	275	1460	3_flowing	2_shallow	NA	0
2012	Nov I	11	late	10	flat	275	1460	3_flowing	2_shallow	NA	0
2012	Nov I	11	late	11	flat	330	1800	2_stagnant	2_shallow	0	1
2012	Nov I	11	late	12	flat	330	1800	2_stagnant	NA	NA	0
2012	Nov I	11	late	13	flat	330	1800	2_stagnant	2_shallow	NA	0
2012	Nov I	11	late	14	flat	330	1800	2_stagnant	1_dry	NA	0
2013	June I	1	early	1	steep	140	755	3_flowing	4_large_deep	1	29
2013	June I	1	early	2	steep	173	780	3_flowing	4_large_deep	NA	0
2013	June I	1	early	3	steep	160	765	3_flowing	4_large_deep	NA	0
2013	June I	1	early	4	steep	148	705	3_flowing	4_large_deep	1	10
2013	June I	1	early	5	steep	151	700	3_flowing	3_small_deep	NA	0
2013	June I	1	early	6	steep	151	700	3_flowing	4_large_deep	NA	NA
2013	June I	1	early	7	steep	151	700	3_flowing	3_small_deep	NA	NA

2013	June I	1	early	8	flat	162	825	3_flowing	3_small_deep	1	13
2013	June I	1	early	9	flat	162	825	3_flowing	3_small_deep	1	11
2013	June I	1	early	10	flat	162	825	3_flowing	3_small_deep	0	1
2013	June I	1	early	11	flat	180	860	3_flowing	3_small_deep	NA	0
2013	June I	1	early	12	flat	180	860	3_flowing	3_small_deep	NA	NA
2013	June I	1	early	13	flat	180	860	3_flowing	3_small_deep	NA	NA
2013	June I	1	early	14	flat	180	860	3_flowing	3_small_deep	NA	0
2013	June II	2	early	1	steep	187	755	3_flowing	4_large_deep	NA	NA
2013	June II	2	early	2	steep	178	745	3_flowing	4_large_deep	NA	0
2013	June II	2	early	3	steep	174	775	3_flowing	4_large_deep	NA	0
2013	June II	2	early	4	steep	155	735	3_flowing	4_large_deep	1	10
2013	June II	2	early	5	steep	151	815	3_flowing	3_small_deep	NA	0
2013	June II	2	early	6	steep	151	815	3_flowing	4_large_deep	NA	NA
2013	June II	2	early	7	steep	151	815	3_flowing	3_small_deep	NA	NA
2013	June II	2	early	8	flat	168	735	3_flowing	NA	NA	0
2013	June II	2	early	9	flat	168	735	3_flowing	3_small_deep	NA	NA
2013	June II	2	early	10	flat	168	735	3_flowing	3_small_deep	NA	0
2013	June II	2	early	11	flat	192	750	3_flowing	3_small_deep	NA	0
2013	June II	2	early	12	flat	192	750	3_flowing	3_small_deep	NA	NA
2013	June II	2	early	13	flat	192	750	3_flowing	3_small_deep	NA	NA
2013	June II	2	early	14	flat	192	750	3_flowing	3_small_deep	NA	0
2013	July I	3	early	1	steep	167	750	3_flowing	4_large_deep	1	25
2013	July I	3	early	2	steep	178	700	3_flowing	4_large_deep	NA	0
2013	July I	3	early	3	steep	192	715	3_flowing	4_large_deep	NA	0
2013	July I	3	early	4	steep	159	710	3_flowing	4_large_deep	1	10
2013	July I	3	early	5	steep	157	655	3_flowing	3_small_deep	NA	0
2013	July I	3	early	6	steep	157	655	3_flowing	4_large_deep	NA	NA
2013	July I	3	early	7	steep	157	655	3_flowing	3_small_deep	NA	NA
2013	July I	3	early	8	flat	173	600	3_flowing	NA	NA	NA
2013	July I	3	early	9	flat	173	600	3_flowing	3_small_deep	NA	NA
2013	July I	3	early	10	flat	173	600	3_flowing	3_small_deep	NA	0
2013	July I	3	early	11	flat	192	720	3_flowing	3_small_deep	NA	0
2013	July I	3	early	12	flat	192	720	3_flowing	3_small_deep	NA	NA

2013	July I	3	early	13	flat	192	720	3_flowing	3_small_deep	NA	NA
2013	July I	3	early	14	flat	192	720	3_flowing	3_small_deep	NA	0
2013	July II	4	early	1	steep	253	810	3_flowing	4_large_deep	NA	NA
2013	July II	4	early	2	steep	208	810	3_flowing	4_large_deep	NA	0
2013	July II	4	early	3	steep	281	785	3_flowing	4_large_deep	NA	0
2013	July II	4	early	4	steep	194	915	3_flowing	4_large_deep	1	15
2013	July II	4	early	5	steep	194	700	3_flowing	3_small_deep	NA	0
2013	July II	4	early	6	steep	194	700	3_flowing	4_large_deep	NA	NA
2013	July II	4	early	7	steep	194	700	3_flowing	3_small_deep	NA	NA
2013	July II	4	early	8	flat	219	735	3_flowing	NA	NA	NA
2013	July II	4	early	9	flat	219	735	3_flowing	3_small_deep	NA	NA
2013	July II	4	early	10	flat	219	735	3_flowing	3_small_deep	NA	0
2013	July II	4	early	11	flat	228	720	3_flowing	3_small_deep	1	22
2013	July II	4	early	12	flat	228	720	3_flowing	3_small_deep	1	38
2013	July II	4	early	13	flat	228	720	3_flowing	3_small_deep	1	5
2013	July II	4	early	14	flat	228	720	3_flowing	3_small_deep	NA	0
2013	Aug I	5	early	1	steep	253	810	3_flowing	4_large_deep	1	32
2013	Aug I	5	early	2	steep	208	810	3_flowing	3_small_deep	NA	0
2013	Aug I	5	early	3	steep	281	785	3_flowing	4_large_deep	NA	0
2013	Aug I	5	early	4	steep	194	915	3_flowing	4_large_deep	NA	NA
2013	Aug I	5	early	5	steep	194	700	3_flowing	3_small_deep	NA	0
2013	Aug I	5	early	6	steep	194	700	3_flowing	4_large_deep	NA	NA
2013	Aug I	5	early	7	steep	194	700	3_flowing	3_small_deep	NA	NA
2013	Aug I	5	early	8	flat	219	735	3_flowing	NA	NA	NA
2013	Aug I	5	early	9	flat	219	735	3_flowing	3_small_deep	1	35
2013	Aug I	5	early	10	flat	219	735	3_flowing	3_small_deep	NA	0
2013	Aug I	5	early	11	flat	228	720	3_flowing	3_small_deep	NA	0
2013	Aug I	5	early	12	flat	228	720	3_flowing	NA	NA	NA
2013	Aug I	5	early	13	flat	228	720	3_flowing	3_small_deep	NA	0
2013	Aug I	5	early	14	flat	228	720	3_flowing	3_small_deep	NA	0
2013	Aug II	6	early	1	steep	NA	NA	3_flowing	4_large_deep	NA	NA
2013	Aug II	6	early	2	steep	NA	NA	3_flowing	3_small_deep	NA	0
2013	Aug II	6	early	3	steep	NA	NA	3_flowing	4_large_deep	NA	0

2013	Aug II	6	early	4	steep	NA	NA	3_flowing	4_large_deep	1	26
2013	Aug II	6	early	5	steep	NA	NA	3_flowing	2_shallow	NA	0
2013	Aug II	6	early	6	steep	NA	NA	3_flowing	4_large_deep	NA	NA
2013	Aug II	6	early	7	steep	NA	NA	3_flowing	3_small_deep	NA	NA
2013	Aug II	6	early	8	flat	255	695	3_flowing	NA	NA	NA
2013	Aug II	6	early	9	flat	255	695	3_flowing	3_small_deep	1	45
2013	Aug II	6	early	10	flat	255	695	3_flowing	3_small_deep	NA	0
2013	Aug II	6	early	11	flat	248	980	3_flowing	3_small_deep	NA	0
2013	Aug II	6	early	12	flat	248	980	3_flowing	NA	NA	NA
2013	Aug II	6	early	13	flat	248	980	3_flowing	3_small_deep	NA	0
2013	Aug II	6	early	14	flat	248	980	3_flowing	3_small_deep	NA	0
2013	Sep I	7	late	1	steep	214	740	3_flowing	4_large_deep	1	80
2013	Sep I	7	late	2	steep	212	710	3_flowing	3_small_deep	NA	0
2013	Sep I	7	late	3	steep	221	730	3_flowing	4_large_deep	NA	0
2013	Sep I	7	late	4	steep	226	740	3_flowing	4_large_deep	1	15
2013	Sep I	7	late	5	steep	247	760	3_flowing	2_shallow	NA	0
2013	Sep I	7	late	6	steep	247	760	3_flowing	4_large_deep	NA	NA
2013	Sep I	7	late	7	steep	247	760	3_flowing	3_small_deep	NA	NA
2013	Sep I	7	late	8	flat	314	840	3_flowing	NA	NA	NA
2013	Sep I	7	late	9	flat	314	840	3_flowing	3_small_deep	1	56
2013	Sep I	7	late	10	flat	314	840	3_flowing	2_shallow	NA	0
2013	Sep I	7	late	11	flat	293	890	3_flowing	3_small_deep	NA	0
2013	Sep I	7	late	12	flat	293	890	3_flowing	NA	0	1
2013	Sep I	7	late	13	flat	293	890	3_flowing	3_small_deep	NA	0
2013	Sep I	7	late	14	flat	293	890	3_flowing	3_small_deep	NA	0
2013	Sep II	8	late	1	steep	235	770	3_flowing	4_large_deep	1	68
2013	Sep II	8	late	2	steep	249	840	3_flowing	3_small_deep	NA	0
2013	Sep II	8	late	3	steep	243	770	3_flowing	3_small_deep	NA	0
2013	Sep II	8	late	4	steep	265	860	3_flowing	4_large_deep	1	12
2013	Sep II	8	late	5	steep	280	860	3_flowing	2_shallow	NA	0
2013	Sep II	8	late	6	steep	280	860	3_flowing	4_large_deep	NA	NA
2013	Sep II	8	late	7	steep	280	860	3_flowing	3_small_deep	NA	NA
2013	Sep II	8	late	8	flat	447	780	3_flowing	NA	NA	NA

2013	Sep II	8	late	9	flat	447	780	3_flowing	3_small_deep	1	52
2013	Sep II	8	late	10	flat	447	780	3_flowing	2_shallow	NA	0
2013	Sep II	8	late	11	flat	343	945	2_stagnant	3_small_deep	NA	0
2013	Sep II	8	late	12	flat	343	945	2_stagnant	NA	NA	NA
2013	Sep II	8	late	13	flat	343	945	2_stagnant	3_small_deep	NA	0
2013	Sep II	8	late	14	flat	343	945	2_stagnant	2_shallow	NA	0
2013	Oct I	9	late	1	steep	398	760	3_flowing	4_large_deep	1	60
2013	Oct I	9	late	2	steep	308	795	3_flowing	3_small_deep	NA	0
2013	Oct I	9	late	3	steep	352	975	3_flowing	2_shallow	NA	0
2013	Oct I	9	late	4	steep	373	1150	3_flowing	4_large_deep	1	12
2013	Oct I	9	late	5	steep	NA	NA	2_stagnant	1_dry	NA	0
2013	Oct I	9	late	6	steep	NA	NA	2_stagnant	4_large_deep	NA	NA
2013	Oct I	9	late	7	steep	NA	NA	2_stagnant	3_small_deep	NA	NA
2013	Oct I	9	late	8	flat	279	NA	3_flowing	NA	NA	NA
2013	Oct I	9	late	9	flat	279	NA	3_flowing	3_small_deep	1	25
2013	Oct I	9	late	10	flat	279	NA	3_flowing	2_shallow	NA	0
2013	Oct I	9	late	11	flat	377	975	2_stagnant	3_small_deep	NA	0
2013	Oct I	9	late	12	flat	377	975	2_stagnant	NA	NA	0
2013	Oct I	9	late	13	flat	377	975	2_stagnant	3_small_deep	NA	0
2013	Oct I	9	late	14	flat	377	975	2_stagnant	2_shallow	NA	0
2013	Oct II	10	late	1	steep	506	1925	2_stagnant	4_large_deep		45
2013	Oct II	10	late	2	steep	444	860	2_stagnant	2_shallow	NA	0
2013	Oct II	10	late	3	steep	512	875	2_stagnant	2_shallow	NA	0
2013	Oct II	10	late	4	steep	601	NA	2_stagnant	4_large_deep	1	39
2013	Oct II	10	late	5	steep	572	NA	2_stagnant	1_dry	NA	0
2013	Oct II	10	late	6	steep	572	NA	2_stagnant	4_large_deep	NA	NA
2013	Oct II	10	late	7	steep	572	NA	2_stagnant	3_small_deep	NA	0
2013	Oct II	10	late	8	flat	261	905	3_flowing	NA	NA	NA
2013	Oct II	10	late	9	flat	261	905	3_flowing	3_small_deep	0	1
2013	Oct II	10	late	10	flat	261	905	3_flowing	2_shallow	NA	0
2013	Oct II	10	late	11	flat	576	2500	2_stagnant	2_shallow	1	3
2013	Oct II	10	late	12	flat	576	2500	2_stagnant	NA	NA	0
2013	Oct II	10	late	13	flat	576	2500	2_stagnant	2_shallow	NA	0

2013	Oct II	10	late	14	flat	576	2500	2_stagnant	1_dry	NA	0
2013	Nov I	11	late	1	steep	NA	NA	1_dry	4_large_deep	1	31
2013	Nov I	11	late	2	steep	603	2500	2_stagnant	2_shallow	NA	0
2013	Nov I	11	late	3	steep	522	1255	2_stagnant	2_shallow	NA	0
2013	Nov I	11	late	4	steep	NA	NA	2_stagnant	3_small_deep	1	43
2013	Nov I	11	late	5	steep	655	1105	2_stagnant	1_dry	NA	0
2013	Nov I	11	late	6	steep	655	1105	2_stagnant	4_large_deep	1	20
2013	Nov I	11	late	7	steep	655	1105	2_stagnant	3_small_deep	NA	0
2013	Nov I	11	late	8	flat	240	860	2_stagnant	NA	NA	NA
2013	Nov I	11	late	9	flat	240	860	2_stagnant	2_shallow	NA	0
2013	Nov I	11	late	10	flat	240	860	2_stagnant	2_shallow	NA	0
2013	Nov I	11	late	11	flat	NA	NA	1_dry	1_dry	NA	0
2013	Nov I	11	late	12	flat	NA	NA	1_dry	NA	NA	0
2013	Nov I	11	late	13	flat	NA	NA	1_dry	2_shallow	NA	0
2013	Nov I	11	late	14	flat	NA	NA	1_dry	1_dry	NA	0
